# Cancer hygiene hypothesis: A test from wild captive mammals

**DOI:** 10.1002/ece3.10547

**Published:** 2023-09-22

**Authors:** Antoine M. Dujon, Jérémy Jeanjean, Orsolya Vincze, Mathieu Giraudeau, Jean‐François Lemaître, Pascal Pujol, Beata Ujvari, Frédéric Thomas

**Affiliations:** ^1^ Geelong, School of Life and Environmental Sciences, Centre for Integrative Ecology Deakin University Waurn Ponds Victoria Australia; ^2^ CREEC/CANECEV (CREES), MIVEGEC, IRD 224–CNRS 5290–Université de Montpellier Montpellier France; ^3^ Institute of Aquatic Ecology, Centre for Ecological Research Debrecen Hungary; ^4^ Evolutionary Ecology Group, Hungarian Department of Biology and Ecology Babes‐Bolyai University Cluj‐Napoca Romania; ^5^ Littoral, Environnement et Sociétés (LIENSs), UMR 7266 CNRS‐La Rochelle Université La Rochelle France; ^6^ CNRS, UMR 5558, Laboratoire de Biométrie et Biologie Evolutive Université de Lyon Villeurbanne France; ^7^ Laboratory of Rare Human Circulating Cells (LCCRH) University Hospital of Montpellier Montpellier France; ^8^ Centre Hospitalier Universitaire Arnaud de Villeneuve Montpellier France

**Keywords:** animal care, biodiversity, captivity, neoplasm, pathogen, treatment

## Abstract

The hygiene hypothesis, according to which the recent reduction of exposure to infectious agents in the human species would be the origin of various diseases, including autoimmune diseases and cancer, has often been proposed but not properly tested on animals. Here, we evaluated the relevance of this hypothesis to cancer risk in mammals in an original way, namely by using information on zoo mammals. We predicted that a higher richness of parasitic cohorts in the species' natural habitat would result in a greater occurrence of evolutionary mismatch due to the reduction of parasites in captive conditions. This, in turn, could contribute to an increased risk of developing lethal cancers. Using a comparative analysis of 112 mammalian species, we explored the potential relationship between cancer risk and parasite species richness using generalized phylogenetic least squares regressions to relate parasite species richness to cancer risk data. We found no strong evidence that parasite species richness increased cancer risk in zoo mammals for any of the parasite groups we tested. Without constituting definitive proof of the irrelevance of the hygienic hypothesis, our comparative study using zoo mammals does not support it, at least with respect to cancer risks.

## Graphical abstract

The hygiene hypothesis suggests that reduced exposure to infectious agents may lead to various diseases, including autoimmune diseases and cancer. To test this hypothesis in relation to cancer risk, a comparative analysis of 112 mammalian species, specifically zoo mammals with reduced exposure to infectious agents, was conducted. The study did not find strong evidence to support the idea that higher parasite species richness increases cancer risk in zoo mammals, indicating that the hygiene hypothesis may not apply to cancer risks at least in this context.

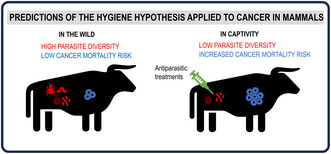

## 1 INTRODUCTION

A leading hypothesis in evolutionary medicine is that many current human pathologies are due to evolutionary mismatches, a phenomenon whereby previously adaptive traits (and their underlying genetic basis) become maladaptive under new environmental conditions, whether spatial and/or temporal (Nesse & Williams, 1994; Stearns, 2012). For example, attraction to sweet flavours and efficient fat storage capacities might be adaptive for humans in ancestral environments where food availability and/or quality were irregular (Leonard, 2007; Neel, 1962), but it is most often maladaptive in industrialized environments with an overabundance of readily available caloric foods. The resulting energy imbalance, coupled with our ancestral propensity to store reserves, now favours weight gain and associated pathologies such as obesity and diabetes (Popkin & Gordon‐Larsen, 2004). Among the long list of contemporary lifestyles responsible for evolutionary mismatches (e.g. reduced sun exposure, lack of exercise, chronic stress and sleep deprivation), a frequently debated one is the loss of our ancestral parasitic cortege, the so‐called “hygiene hypothesis” proposed more than 30 years ago (Pfefferle et al., 2021; Strachan, 1989). Indeed, while throughout their evolutionary history humans have always lived in close vicinity with a large number of pathogens from their natural environment (parasites, bacteria, viruses and fungi), these contacts have recently been strongly reduced in many geographic areas owing to marked environmental or sociological changes (Thomas et al., 2012). This has been undoubtedly associated with many health benefits given the detrimental effects of infectious diseases. Yet, reducing parasitic contact has also had negative consequences. This is because we have co‐evolved with these parasitic organisms, and in doing so, we have created complex interactions that are not easily terminated without having a negative impact on the host's health and fitness. Helminths, for example, have been massively eradicated with anti‐helminthic medications from industrialized countries, and their absence seems to be now involved in a range of hyperinflammatory pathologies, including irritable bowel disease, multiple sclerosis, Crohn's disease, allergies, type 1 diabetes and asthma (Bach, 2018; Fleming, 2011; Parker & Ollerton, 2013). The fact that symptoms of Crohn's disease can be ameliorated by ingesting eggs of a porcine nematode (e.g. *Trichuris suis*, which hatches but does not grow in humans, see, Summers et al., 2005) opens up prospects for possible applications of evolutionary medicine in the treatment of some modern health disorders (Fernandes et al., 2019; Greb & Gottlieb, 2013; Summers et al., 2003).

Cancers are also on the list of diseases whose initiation and/or progression may – at least to some extent – result from evolutionary mismatches (Aktipis & Nesse, 2013). For instance, overeating and obesity (e.g. Avgerinos et al., 2019), population migration changing levels of sunlight exposure (e.g. Jablonski, 2014), smoking (e.g. Alexandrov et al., 2016), reduced physical activity (e.g. Jurdana, 2021), night‐time light pollution (e.g. Anisimov et al., 2013) and changes in the number of reproductive cycles in women (e.g. Aktipis et al., 2015; Bieuville et al., 2023) are all examples in a long list of factors that have substantially changed in modern countries and that have also been shown to exacerbate malignant cell dynamics. Surprisingly, few studies have explored the relevance of the hygiene hypothesis for cancers (Garn et al., 2021). Among them, Greaves proposed the “delayed infection hypothesis” as a possible explanation for childhood acute (lymphoblastic/myeloid) leukaemia, which typically peaks at 2–5 years of age in developed countries (Greaves, 2006, 2018). The onset of this disease is thought to occur in two stages. The first is the appearance, before birth, of a pre‐leukaemic clone due to a chromosomal translocation or hyperdiploidy. If during childhood, a deletion or genetic mutation also occurs, this leads to leukaemia. Greaves proposed that children exposed to a rich microbial environment early in life, as in ancestral environments, have a greater ability to prevent the second stage, and therefore would not develop leukaemia. According to this argument, early exposure to infectious agents would allow the immune system to mature more quickly and thus better fight an emerging leukaemia if the second genetic defect occurs. On the other hand, susceptible children whose immune systems remain naive due to lack of contact with “old friends” during the early years of life may not be able to eradicate expanding clones of leukaemia cells (but see Cardwell et al., 2008; Simpson et al., 2007). Also consistent with the hygiene hypothesis, a recent meta‐analysis showed that living on a farm with contact with animals and pets reduced the odds of child development lymphoblastic leukaemia between 8% and 35% depending on the type of animals, in addition to several allergies (Orsi et al., 2018).

We propose here to investigate the hygienic hypothesis of cancers through original comparative analysis by exploring the possible links between the probability of occurrence and/or death of cancers in captive mammals in relation to the richness of their pathogenic community when living in their natural ecosystems. Indeed, a characteristic shared by virtually all zoo animals is that they are, for a variety of reasons, cleared of a fraction of their parasitic load (Hosey et al., 2009). This provides a unique experimental context for exploring the hygiene hypothesis of pathologies, including cancer (Dujon, Schofield, et al., 2021). Several mechanisms suggest infections by parasites may offer protection from cancer in mammals. This includes, among others, the suppression of inflammation, the promotion of antitumour immunity or the alteration of the tumour microenvironment, preventing cancer progression (see Oikonomopoulou et al., 2013). Helminths, for example, are relatively well documented in mammals and look particularly promising considering their positive effect on autoimmune diseases in humans (Fernandes et al., 2019; Greb & Gottlieb, 2013; Stephens et al., 2017; Summers et al., 2003, 2005). Under this hypothesis, we predict that animals with high parasite diversity in the wild, either overall or disaggregated by major parasite groups, should experience the greatest parasite mismatch when in the zoo, which should, all else being equal, exacerbate their cancer risk.

## 2 MATERIALS AND METHODS

### 2.1 Cancer mortality risk dataset

We obtained cancer mortality risk data from Vincze et al. (2022). This study used data provided by Species360 and the Zoological Information Management System (ZIMS), an international nonprofit organization that maintains a centralized, real‐time database of animals in human care (aggregating information from over 1200 zoos worldwide). They relied on the high probability of body retrieval of deceased zoo animals and postmortem pathological records, which allows to detect and document cancer‐related mortality (although with lower probability in case of liquid tumours, early‐stage cancers or small, but lethal tumours; see the original publication for details). Cancer is only registered in this database for deceased animals and only if the inspecting veterinary pathologist considered it to be a factor that contributed to the individual's death. Only species in which postmortem pathological records were available for at least 20 adult individuals, irrespective of the cause of death, were included in their analyses. Vincze and colleagues also excluded all species that were subject to domestication as well as their wild ancestors, as domestication is widely regarded as a major contributing factor to inbreeding depression and higher risk of developing cancer (Thomas et al., 2020). In addition, cancer mortality risks provided by Vincze et al. were calculated by pooling both male and female data.

Vincze et al. calculated two metrics of cancer risk, namely the cancer mortality risk (CMR) and incidence of cancer mortality (ICM). They estimated the cancer mortality risk as the ratio between the number of cancer‐related deaths and the total number of individuals whose postmortem pathological records were entered in the database, which can be used to perform complete case analysis. Incidence of cancer mortality, which is a metric of cancer mortality risk eliminating potential biases due to disregarding left truncation (i.e. cancer before individuals enter the study) and right censoring (individuals alive, thus with unknown fate at data extraction), was computed using a Kaplan–Meier estimator (see Vincze et al., 2022 for full details on the calculation of this metric). The ICM was computed for species in which survival is correctly estimated until old ages (i.e. data allowing the estimation of age‐specific survival until the age at which only 10% of individuals are surviving). Thus, if parasite species richness has a strong effect on cancer risk, it should be observable on both metrics, but with different effect sizes (in many cases, effect sizes are reduced once confounding factors are accounted for). Finally, Vincze et al. considered species to have cancer if at least one individual with postmortem pathological record was diagnosed with cancer, a classification we used in our study too.

### 2.2 Parasites dataset

We obtained raw data on parasites' incidence in mammals from the global mammal parasite species database (version 2.0); a database compiling information on the diversity of parasites found in wild ungulates (artiodactyls and perissodactyls), carnivores and primates (Stephens et al., 2017). We retrieved parasite's incidence information for 112 species for which cancer incidence data were already available. We then estimated parasitic species richness using the chao2 estimator (Chao & Chiu, 2016) using species incidence and the number of publications included in the database as sampling effort. Parasite species richness was calculated for species with at least 2, 6 and 14 publications in the database (*n* = 97, 58 and 31 mammal species, respectively). Those three estimates allow us to account for the effect of sampling effort on parasitic species richness. Based on extensive simulations (Teitelbaum et al., 2020), this approach was found to be the best for estimating parasitic species richness in the global mammal parasite species database. In addition, for each mammal species, we computed the species richness of not only arthropods, helminths, bacteria, protozoa and viruses but also microparasites (i.e. viruses, bacteria and protozoa) and macroparasites (i.e. arthropods and helminths). Microparasites typically reproduce within the host while macroparasites reproduce outside the host (Fenton, 2008). A range of microparasites, especially viruses (Madsen et al., 2017), are linked or suspected to cause cancer in wildlife (e.g. in sea turtles, (Jones et al., 2016), pikes (Papas et al., 1976), newts (Asashima et al., 1982) and Western barred bandicoots (Woolford et al., 2009)) and thus we predict that removing them would decrease the cancer risks associated with those infections. Some helminth species are known to promote cancer in humans (e.g. liver and blood flukes, Brindley et al., 2015) and it may also be the case in wildlife (Madsen et al., 2017). However, it is also possible that the presence of helminths stimulates the immune system, reducing cancer risk (as predicted by the hygiene hypothesis). Parasites causing chronic inflammation, a tumour promotor, may also increase cancer risk (Oikonomopoulou et al., 2013).

### 2.3 Confounding variables

Vincze et al. (2022). showed that a diet consisting primarily of vertebrates increased cancer mortality risk and thus should be considered as a potential confounding variable. Here, we consider that diet is part of the cancer risk landscape of animals in which multiple risk factors increase the probability of developing cancer during their lifetime (see Dujon, Aktipis, et al., 2021). We therefore reused Vincze et al. classification, in which each mammal species is binary classified as primarily eating vertebrate or not, as initially proposed by Kissling et al. (2014) (*n* = 190). Missing information on the diet of one species, the Californian sea lion (*Zalophus californianus*, classified as primarily eating vertebrates, and in which cancer was detected, Weise & Harvey, 2008), was manually added to the dataset. Vincze and colleagues also observed a trend between cancer risk and body mass or lifetime expectancy (negative and positive trends, respectively), and we thus tested the effect of those variables in our analyses.

### 2.4 Phylogenetic analyses

We used phylogenetic generalized least‐squares (PGLS) regressions to quantify the effect of the parasite species richness on the cumulative cancer mortality risk of mammal species following a two‐step protocol (first using binary PGLS, then continuous PGLS). We first investigate the potential effect of parasite species richness to increase the probability of detecting cancer and then quantify their effect on the CMR and ICM of a species. Vincze et al. (2022) showed that the probability of detecting cancer mortality in a species depended on the number of animals with a pathological record (i.e. the sample size) and this thus should be included in the models. Here, we used binary PGLS models in order to investigate whether cancer mortality in captive populations was more likely to be detected in species with a higher parasite species richness in their natural environment.

To account for the lack of independence between species due to shared ancestry, we reused the phylogenetic tree from Vincze et al. (2022). This tree was created by obtaining a rooted consensus from a sample of 1000 equally plausible phylogenetic trees published by Upham et al. (2019). Since the precision of the cancer mortality estimates is affected by the sample size used to compute them (Vincze et al., 2022), we included the log of the number of animals with a necropsy record available as an explanatory variable in the binary PGLS, and also used that number to weight each species in univariate and multivariable continuous PGLS. In addition, we logit‐transformed cancer mortality, or species richness values prior to fitting models to ensure model residual normality and homogeneity of variance (on the link function; Warton & Hui, 2011). When required we computed the models' marginal effects to visualize how a variable affected the cancer mortality of mammals (Mize et al., 2019).

All analyses were performed using R (R Development Core Team, 2020; Version 4.0.2). Phylogenetic analyses were computed using the “nlme” and “ape” packages (Paradis & Schliep, 2019; Pinheiro et al., 2021).

## 3 RESULTS

### 3.1 Effect of parasite species richness on the probability of detecting cancer mortality

The screening of potential confounding variables revealed no significant effect on the body mass (beta = −0.000717 ± 0.001536 SE, *p* = .641, *n* = 97 species), life expectancy (beta = 0.000308 ± 0.000172 SE, *p* = .073, *n* = 97 species) and diet (beta = 0.070845 ± 0.970743 SE, *p* = .942, *n* = 97 species) on the probability of detecting cancer mortality. Thus, they were not included in subsequent binomial analyses.

**FIGURE 1:**
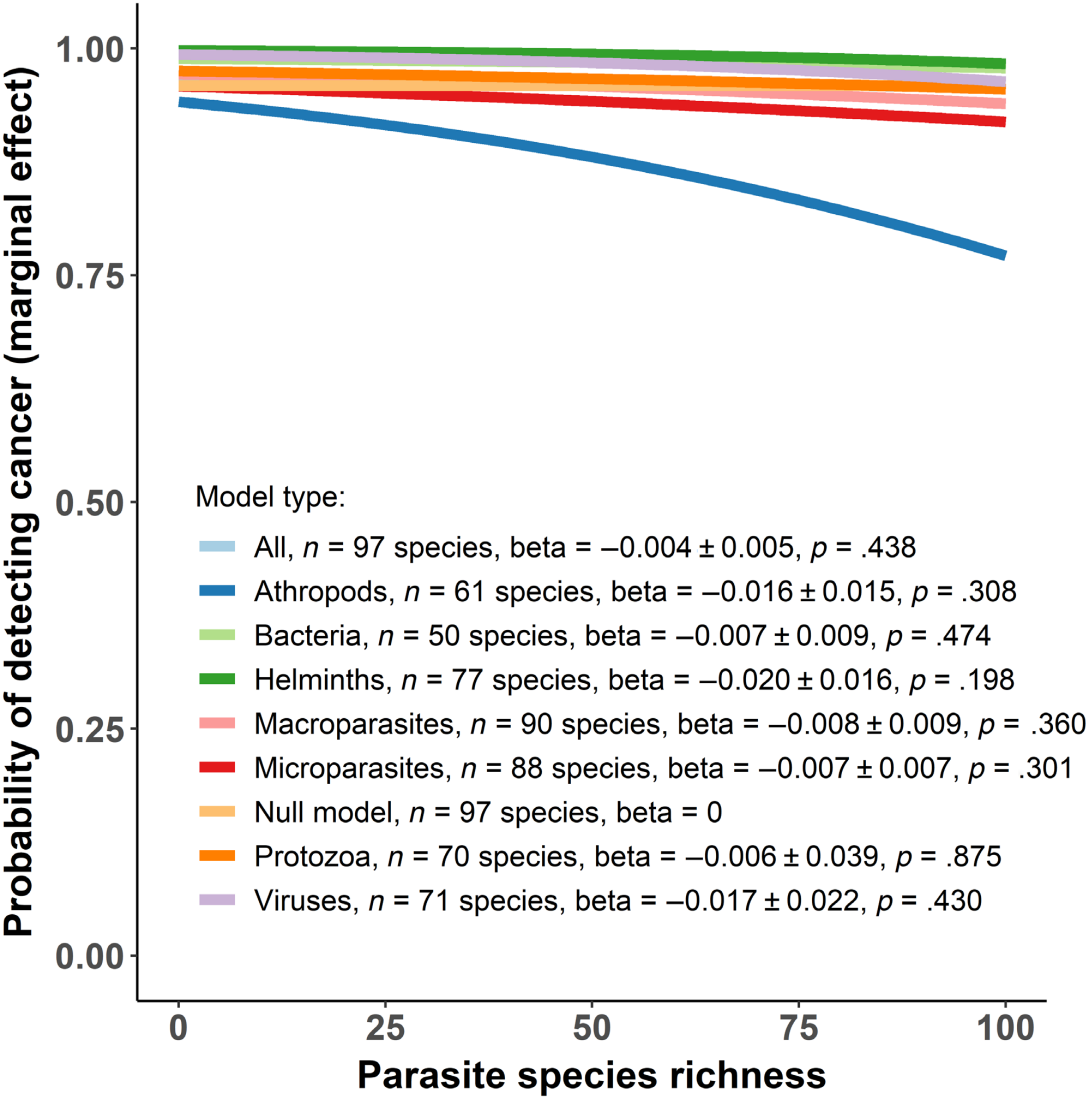
Lack of effect of parasitic species richness on the probability of detecting tumours during autopsy of zoo animals. Mammalian species with high parasite species richness in the wild do not have an increased risk of developing detectable tumours when raised in capivity. A sample of 100 necropsied individuals was used to calculate marginal effects. The null model quantifies the probability of detecting tumours at autopsy for a sample size of 100 individuals (sample size is significant in all models). A breakdown of the number of mammalian species that were used to fit each model (*n* ≥ 2 publications in the Global Mammal Parasite Database) is provided along with the slope (beta) between parasite species richness and the probability of detecting cancer at necropsy.

We found no significant effect of parasite species richness on the probability of detecting cancer mortality in zoo mammals. This was the case even when parasites were divided into different groups (see Figure 1 for detailed statistics). Furthermore, this lack of relationship was also observed when mammalian species were divided into primate (*n* = 31 species), carnivore (*n* = 26 species) or artiodactyl (*n* = 37 species) groups, or were restricted to mammalian species with ≥2 publications in the Global Mammalian Parasite Database (*n* ≥ 6 and *n* ≥ 14 publications, see Table S1–S3 and Figures S1 and S2).

### 3.2 Effect of parasite species richness on cancer mortality risk of zoo mammals

The screening of potential confounding variables indicated that diet had a significant effect on the cancer mortality risk (Beta_CMR_ = 0.66226 ± 0.293481 SE, *p* = .027, *n* = 72 species) and incidence of cancer mortality (Beta_ICM_ = 0.944908 ± 0.346630 SE, *p* = .008, *n* = 66 species) with species primarily eating vertebrates being under increased risk for both metrics. This was not the case for body mass (Beta_CMR_ = −0.000258 ± 0.0006417 SE, *p* = .689, *n* = 72 species; Beta_ICM_ = −0.000703 ± 0.000824 SE, *p* = .397, *n* = 66 species) and life expectancy (Beta_CMR_ = 0.000087 ± 0.000065 SE, *p* = .184, *n* = 72 species; Beta_ICM_ = 0.000115 ± 0.000093 SE, *p* = .221, *n* = 66 species). Thus, diet was included as a confounding variable in all the subsequent analyses.

**FIGURE 2:**
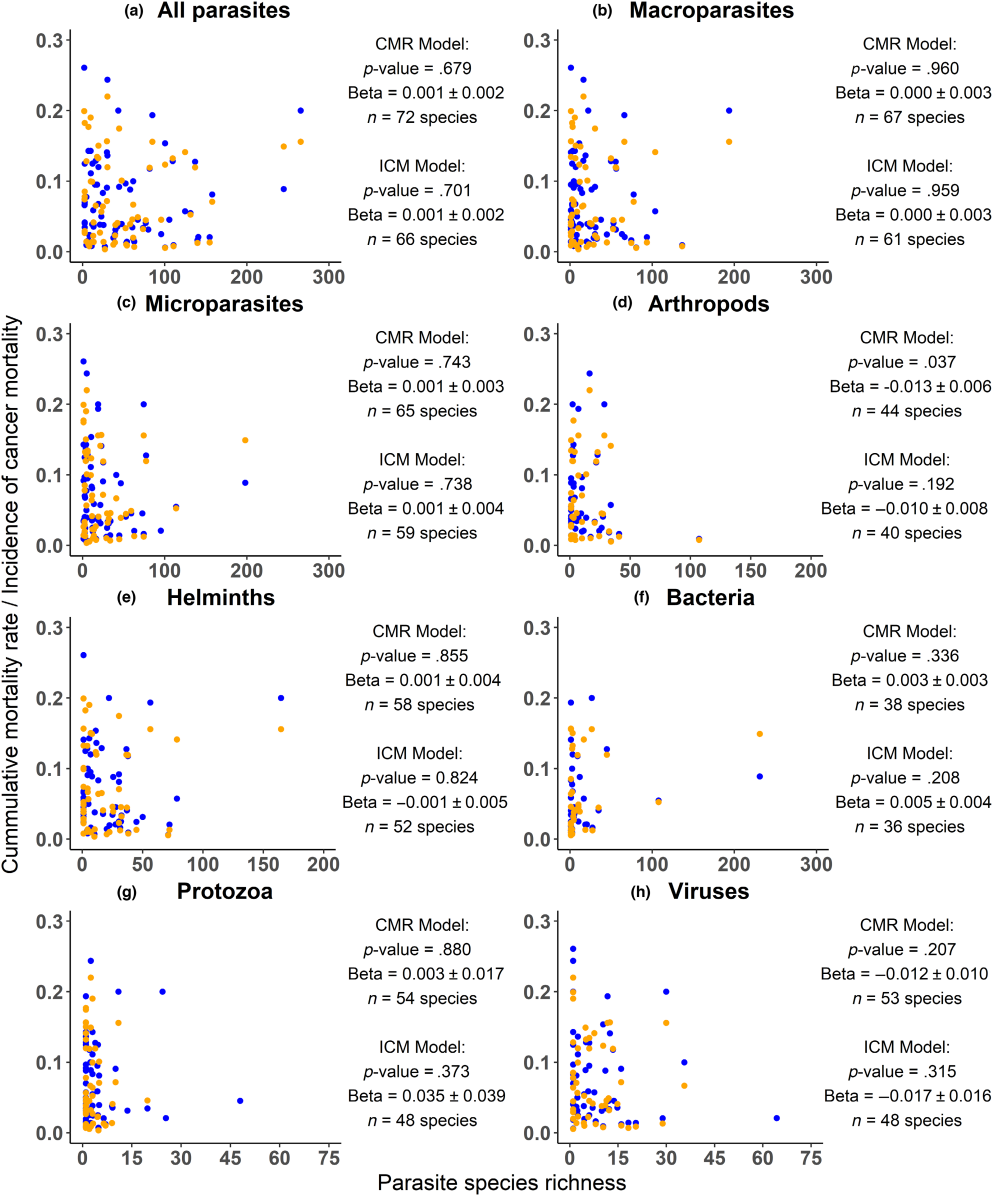
Cancer mortality rate (blue) and incidence of cancer mortality (orange) of captive mammal species as a function of their estimated parasite species richness in the wild. The *p*‐value, beta slope between cancer mortality rate/incidence of cancer mortality and parasitic species richness, and the number of species included in the continuous PGLS models are shown for each parasite group. Only mammalian species with at least ≥2 publications available in the global mammal parasite database are included in this analysis.

For species with ≥2 publications in the global mammal parasite database, we found no significant effect of parasite species richness on cancer mortality rate or incidence of cancer mortality of captive mammals, regardless of how the parasites were pooled (see Figure 2 for detailed statistics). Similarly, no significant trend was observed when log transforming the parasite species richness to account for outliers (Figure S5).

**FIGURE 3:**
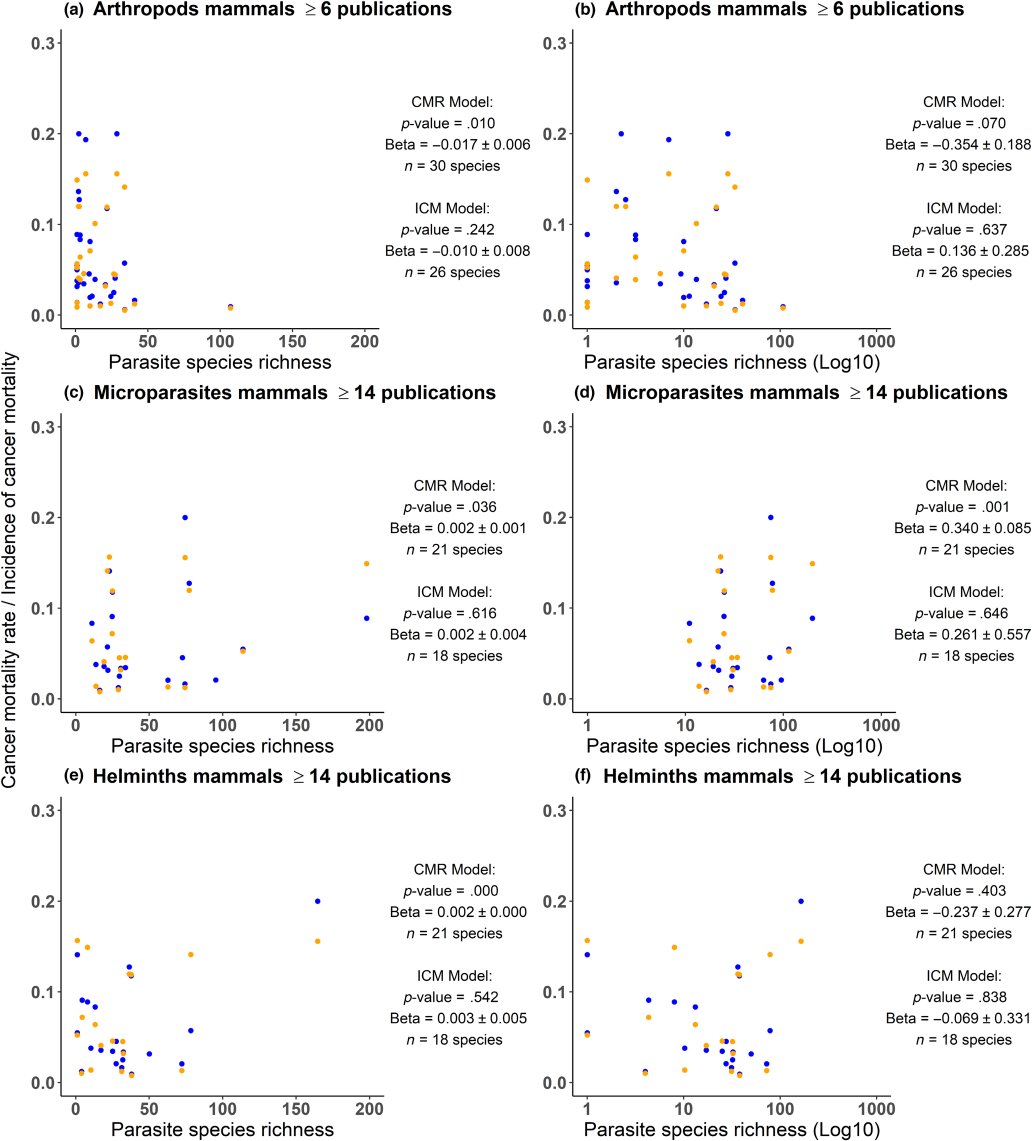
Cancer mortality rate (blue) and incidence of cancer mortality (orange) of captive mammal species as a function of their estimated parasite species richness (in the wild) with special focus on (a, b) arthropods for mammal species with ≥6 publications in the global mammal parasite database (c, d), microparasites (e, f) and helminths for mammal species with ≥14 publications in the global mammal parasite database. Significant trends, when they are not driven by outliers, are only observed between parasite species richness and cancer mortality risk, but not the incidence of cancer mortality which can be considered as being a more accurate measurement of cancer risk. There is thus limited support for a link between parasite species richness and cancer risk.

For species with ≥6 publications in the global mammal parasite database, a significant positive trend was observed between the species richness of arthropods and the cancer mortality rate of mammals but not the incidence of cancer mortality (Figure 3a,b). However, that positive trend was driven by outliers and became nonsignificant if the species richness was log‐transformed to account for the data overdispersion. No other significant association between parasite species richness and cancer mortality rate or incidence of cancer mortality was observed for the other parasitic groups (Figures S3 and S6).

For species with ≥14 publications in the global mammal parasite database, a significant positive trend was observed between the microparasite species richness and the cancer mortality rate of mammal species but not the incidence of cancer mortality. The trend observed in the cancer mortality rate remained significant when the species richness was log‐transformed (Figure 3c,d). No other association among parasite species richness, cancer mortality rate and incidence of cancer mortality was observed for the other parasitic groups (see Figures S4 and S7).

Regardless of how the data were analysed, we found no significant interactions between the diet and the cancer mortality risk or incidence of cancer mortality.

## 4 DISCUSSION

Although the “hygiene hypothesis” is frequently presented as a plausible explanation for many modern human diseases, this hypothesis has never been explored across species. Because zoo animals are often cleared of a significant fraction of their parasitic load and monitored for malignant diseases (Hosey et al., 2009), they provide an interesting context for assessing the extent to which parasite‐based evolutionary mismatch exacerbates cancer dynamics. We predicted that all else being equal, there should be a positive relationship between the richness of parasite a community of animal harbours in the wild and their vulnerability to cancer problems when in captive, parasite‐free, conditions. Conversely to this expectation, we found very limited evidence of an association between known parasitic richness in wild mammals and their respective probability of detecting cancer mortality, their cancer mortality risk and their incidence of cancer mortality when in zoo conditions. Thus, our results do not support the cancer hygiene hypothesis, but some limitations must be discussed.

First, we cannot rule out the existence of small effect sizes; however, they cannot be detected with the approach we used (and would require a larger sample size). Secondly, it is also possible that only few parasite species are actively involved in the hygiene hypothesis so considering the entire parasitic richness adds statistical noise and prevents us from detecting existing effects. While being a great resource, the data coverage of the global mammals' parasite database lacks the resolution to explore individual species of pathogens (e.g. a number of cancer‐causing parasites are documented in mammals; Erdélyi et al., 2009; Woolford et al., 2009) and a range of other taxa (Dujon, Ujvari, & Thomas, 2021), which is why we relied on a species richness estimation.

Another interpretation of the observed lack of effect is that, although reduced, the parasite load is never completely eliminated in zoo animals. Helminths, arthropods or viruses are commonly observed in zoo animals. The dataset used in this study is an aggregate of multiple zoos, each of them likely to have variations in the level of care to animals and the proportion of parasites removed (Adler et al., 2011; Adrus et al., 2019; Lim et al., 2008; Mir et al., 2016; Panayotova‐Pencheva, 2013; Vonfeld et al., 2022). The presence of these parasites in captive populations may be sufficient to prevent an increased risk of cancer mortality that a complete elimination of the parasite load would cause. Alternatively, we cannot exclude that the cancer hygiene hypothesis is relevant, but predicted associations cannot be detected in this analysis because captive conditions would concomitantly provide mammals with better abilities to fight emerging cancers (e.g. absence of predators or fights with competitors from other species, Hosey et al., 2009). Since cancer dynamics are at a crossroad and involve many trade‐offs (Jacqueline et al., 2017), we cannot exclude that compensatory mechanisms boosting cancer defences hide higher cancer risks, with a net effect being an apparent absence of relationship (see also Dujon et al., 2023). For instance, the relatively high food quality provided by zoological institutions might buffer the trade‐off among growth, reproduction and immuno‐competence in the other hand, which could ultimately constitute an additional protection against cancer. The inclusion of care information in future studies may allow us to determine which of those hypotheses explain the cancer risk patterns (or lack of) in zoo animals; a valuable information to manage cancer risk in conservation programmes (Hamede et al., 2020).

The approach we used to quantify parasite species diversity, despite being one of the most appropriate for the global database of mammalian parasites, likely underestimates true parasite species richness (see extensive simulations in Teitelbaum et al., 2020). It is possible that as sampling effort increases, the precision of a mammal parasite species richness will improve, potentially detecting small effects of parasite species richness on cancer risk. It may be the case for microparasites as we observed a positive association with species richness and cancer mortality rate (but driven by outliers). However, our results suggest that these effects would be relatively small, otherwise, they would have been detected when analysing both the cancer mortality rate and the more accurate incidence of cancer mortality metric. It is also worth mentioning that the majority of mammal species are not present in zoos, or have their parasite diversity quantified (Brereton & Brereton, 2020; Burgin et al., 2018). Our study used some of the best openly available datasets on parasite diversity and cancer risk in wild ungulates, carnivores and primates (Stephens et al., 2017; Vincze et al., 2022). We expect the size of databases to increase, and we encourage scientists to reanalyse them in a few years once they are expanded upon.

In this study, we tested the cancer hygiene hypothesis at the inter‐specific level. Our analysis does not exclude the possibility that for a given mammal species, interindividual variations in parasite species richness are associated with an increased cancer risk. This cannot be tested with the dataset we used as it would require data from multiple populations (or a breakdown between different zoos). It is not uncommon to observe spatiotemporal variability in cancer prevalence in wildlife populations (see, for example, the case of green sea turtles, Dujon, Ujvari, & Thomas, 2021, gonadal neoplasia in marine bivalves, Carballal et al., 2015, and liver neoplasm in marine fish, Myers et al., 1990). Very similarly our study used cancer mortality risk and incidence of cancer mortality aggregated from all cancer types. Our findings do not exclude the possibility that the risk of developing certain cancer types (e.g. liquid tumours as observed in leukaemia in humans), rather than all cancers, increases when the parasitic load is removed. Our study also does not cover benign tumours because the cancer mortality metrics we use are estimated using animals for which cancer was considered to be a factor that contributed to the individual's death (which implies malignancy).

Finally, our study highlights the importance of testing key hypotheses about cancer ecology and evolution, such as the hygiene hypothesis, on a wide range of species. Indeed, these hypotheses have often been developed from data on human cancers, which does not necessarily mean that they apply to all mammalian species. This type of study helps to investigate whether there are general patterns governing cancer risk in mammals or, alternatively, whether a species‐by‐species approach is needed to explain differences in cancer susceptibility.

## AUTHOR CONTRIBUTIONS

**Antoine M. Dujon:** Conceptualization (equal); data curation (equal); formal analysis (equal); investigation (equal); methodology (equal); project administration (equal); resources (equal); software (equal); validation (equal); visualization (equal); writing – original draft (equal); writing – review and editing (equal). **Jérémy Jeanjean:** Validation (supporting); writing – review and editing (supporting). **Orsolya Vincze:** Data curation (equal); validation (equal); writing – review and editing (supporting). **Mathieu Giraudeau:** Writing – review and editing (supporting). **Jean‐François Lemaître:** Conceptualization (equal); validation (equal); writing – review and editing (equal). **Pascal Pujol:** Writing – review and editing (supporting). **Beata Ujvari:** Funding acquisition (equal); writing – review and editing (supporting). **Frédéric Thomas:** Conceptualization (equal); funding acquisition (equal); project administration (equal); writing – original draft (equal); writing – review and editing (equal).

## FUNDING INFORMATION

This work was funded by the MAVA Foundation and the following grants: ANR TRANSCAN (ANR‐18‐CE35‐0009), a CNRS International Associated Laboratory Grant and an Alfred Deakin Postdoctoral Research Fellowship (AMD). OV is supported by the National Scientific Research Fund (OTKA K143421) and by a grant of the Romanian Ministry of Research, Innovation and Digitization (CNCS ‐ UEFISCDI, project number PN‐III‐P1‐1.1‐TE‐2021‐0502).

## CONFLICT OF INTEREST STATEMENT

The authors declare no conflict of interest.

## Supporting information


Data S1

## Data Availability

The Global Mammal Parasite Database dataset is available at https://parasites.nunn‐lab.org/. The cancer risk dataset is available as Supporting Information from Vincze et al. (2022).
