# Supplementary material for: Cancer hygiene hypothesis: A test from wild captive mammals
Source: Ecol Evol. 2023 Sep 22;13(9):e10547. doi: 10.1002/ece3.10547 (PMC10515881; doi:10.1002/ece3.10547)
Supplement: Supplementary file 1 — Data S1 [file ECE3-13-e10547-s001.docx]

# Cancer hygiene hypothesis: a test from wild captive mammals

**Supplementary Materials**

# Antoine M. Dujon^1,2^, Jérémy Jeanjean ^2^, Orsolya Vincze^3,4,5^, Mathieu Giraudeau^5^, Jean-François Lemaître^6^, Pascal Pujol^2,7^, Beata Ujvari^1,2^ & Frédéric Thomas^2^

^1^Deakin University, Geelong, School of Life and Environmental Sciences, Centre for Integrative Ecology, Waurn Ponds, Vic 3216, Australia.

^2^ CREEC/CANECEV (CREES), MIVEGEC, IRD 224–CNRS 5290–Université de Montpellier, Montpellier, France

^3^ Institute of Aquatic Ecology, Centre for Ecological Research, Debrecen, Hungary

^4^ Evolutionary Ecology Group, Hungarian Department of Biology and Ecology, Babes-Bolyai University, Cluj-Napoca, Romania

^5^ Littoral, Environnement et Sociétés (LIENSs), UMR 7266 CNRS-La Rochelle Université, La Rochelle, France

^6^ CNRS, UMR 5558, Laboratoire de Biométrie et Biologie Evolutive, Université de Lyon, Université Lyon 1, Villeurbanne, France,

^6^ Laboratory of Rare Human Circulating Cells (LCCRH), University Hospital of Montpellier, Montpellier, France

^7^ Centre Hospitalier Universitaire Arnaud de Villeneuve, Montpellier, France

Corresponding author: antoine.dujon@yahoo.fr

*Effect of parasite species richness on the probability of detecting tumours during necropsy*

**Supplementary Table 1,2,3:** Effect of the parasitic species richness of various parasites groups in the probability of detecting cancer in primates, carnivora and artiodactyls for which at least two publications were available in the global mammalian parasite database. No statistically significant relationships were detected.

| Primates | | | |  |
| --- | --- | --- | --- | --- |
|  | | | |  |
| Type | N mammals | Slope±SE | P-value |  |
| All | 31 species | beta = 0.093±0.053 | p = 0.081 |  |
| Macroparasites | 27 species | beta = 0.119±0.098 | p = 0.222 |  |
| Microparasites | 27 species | beta = 0.066±0.033 | p = 0.05 |  |
| Arthropods | 13 species | beta = -0.048±0.27 | p = 0.86 |  |
| Helminths | 25 species | beta = 0.245±0.243 | p = 0.314 |  |
| Protozoa | 24 species | beta = 0.321±0.272 | p = 0.237 |  |
| Viruses | 19 species | beta = 0.184±0.159 | p = 0.247 |  |

Note: All primates species for which bacteria species richness was estimated developed cancer, which prevented the fitting of a binomial PGLS model.

| Carnivora | | | |  |
| --- | --- | --- | --- | --- |
|  | | | |  |
| Type | N mammals | Slope±SE | P-value |  |
| All | 26 species | beta = -0.007±0.007 | p = 0.341 |  |
| Macroparasites | 24 species | beta = -0.005±0.013 | p = 0.723 |  |
| Microparasites | 26 species | beta = -0.016±0.008 | p = 0.060 |  |
| Arthropods | 18 species | beta = 0.265±0.236 | p = 0.260 |  |
| Helminths | 19 species | beta = -0.004±0.016 | p = 0.792 |  |
| Bacteria | 14 species | beta = -0.009±0.009 | p = 0.271 |  |
| Protozoa | 20 species | beta = -0.281±0.787 | p = 0.721 |  |
| Viruses | 23 species | beta = -0.02±0.067 | p = 0.764 |  |

| Artiodactyla | | | |  |
| --- | --- | --- | --- | --- |
|  | | | |  |
| Type | N mammals | Slope±SE | P-value |  |
| All | 37 species | beta = -0.006±0.007 | p = 0.369 |  |
| Macroparasites | 36 species | beta = -0.013±0.012 | p = 0.287 |  |
| Microparasites | 33 species | beta = -0.005±0.01 | p = 0.628 |  |
| Arthropods | 28 species | beta = -0.016±0.016 | p = 0.315 |  |
| Helminths | 30 species | beta = -0.079±0.051 | p = 0.123 |  |
| Bacteria | 27 species | beta = 0±0.021 | p = 0.984 |  |
| Protozoa | 24 species | beta = -0.402±0.278 | p = 0.148 |  |
| Viruses | 28 species | beta = -0.027±0.032 | p = 0.402 |  |

**
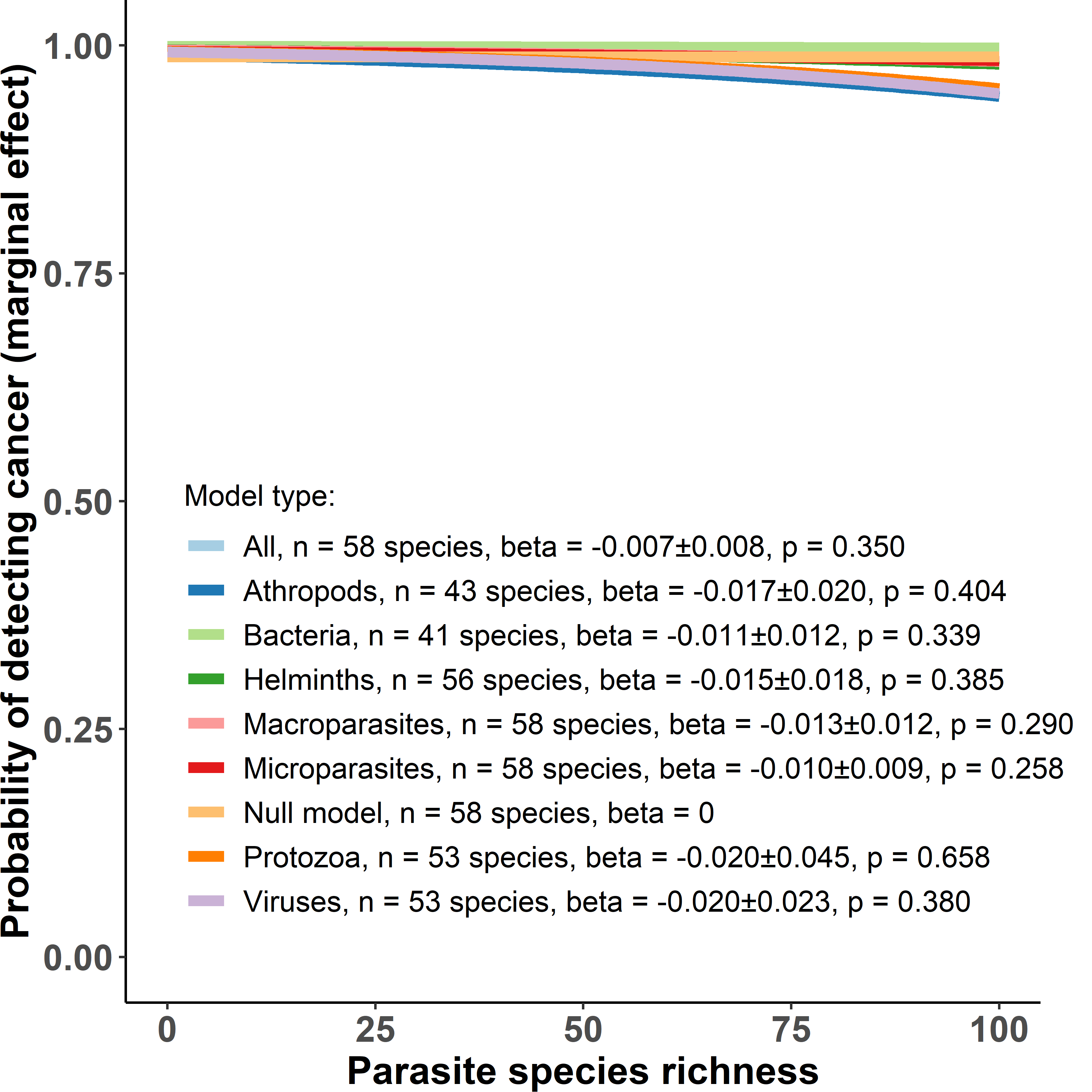
**

**Supplementary Figure 1:** Illustration of the lack of effect of parasitic species richness on the probability of detecting tumours during autopsy of zoo animals. Mammalian species with high parasite species richness do not have an increased risk of developing detectable tumours. A sample of 100 necropsied individuals was used to calculate marginal effects. The null model quantifies the probability of detecting tumours at autopsy for a sample size of 100 individuals (sample size is significant in all models). A breakdown of the number of mammal species that were used to fit each model (n>6 publications in the global mammal parasite database) is provided along with the slope (beta) between parasite species richness and the probability of detecting cancer at necropsy.


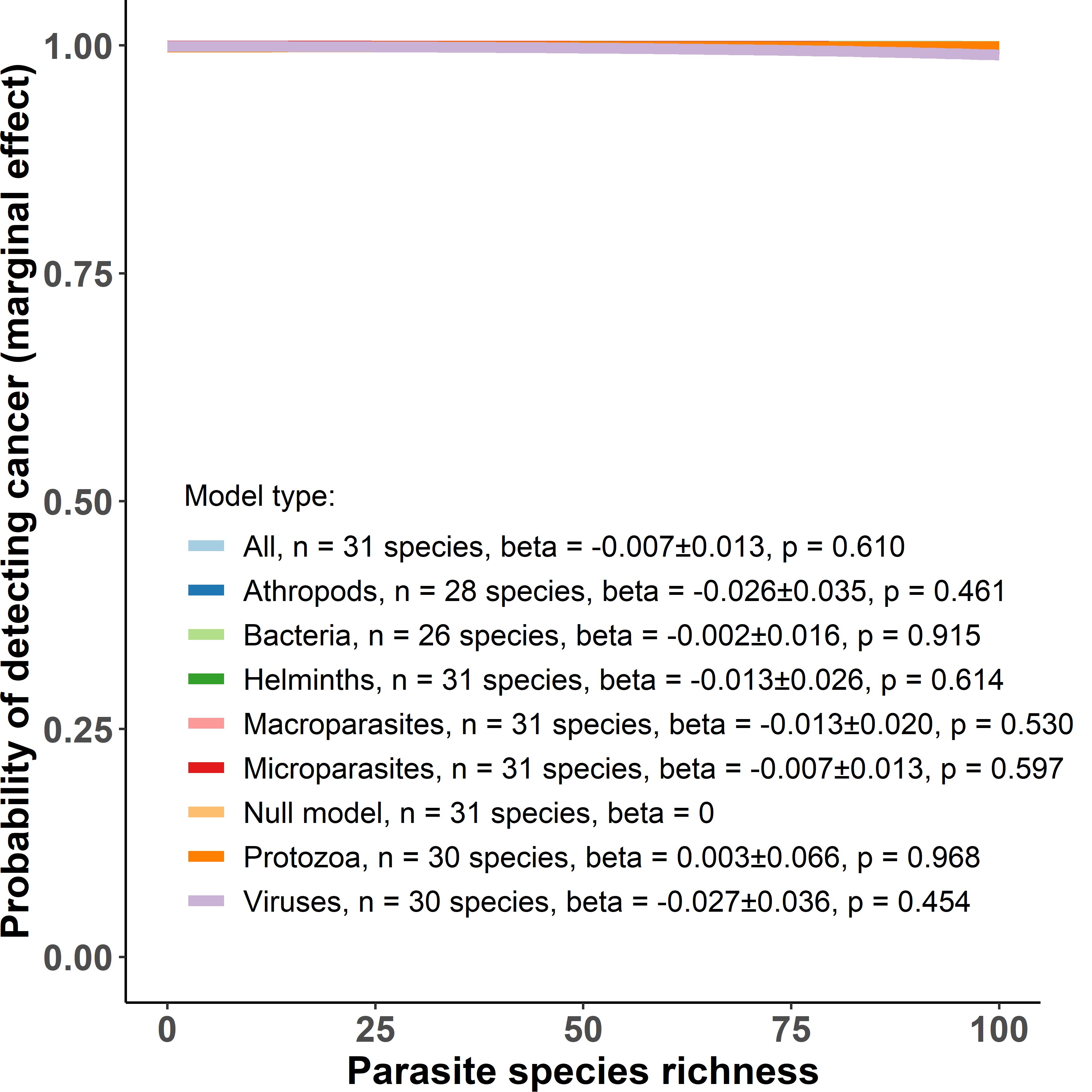


**Supplementary Figure 2:** Illustration of the lack of effect of parasitic species richness on the probability of detecting tumours during autopsy of zoo animals. Mammal species with high parasite species richness do not have an increased risk of developing detectable tumours. A sample of 100 necropsied individuals was used to calculate marginal effects. The null model quantifies the probability of detecting tumours at autopsy for a sample size of 100 individuals (sample size is significant in all models). A breakdown of the number of mammal species that were used to fit each model (n>14 publications in the global mammal parasite database) is provided along with the slope (beta) between parasite species richness and the probability of detecting cancer at necropsy.

*Effect of parasite species richness on cancer mortality risk of zoo mammals*

*
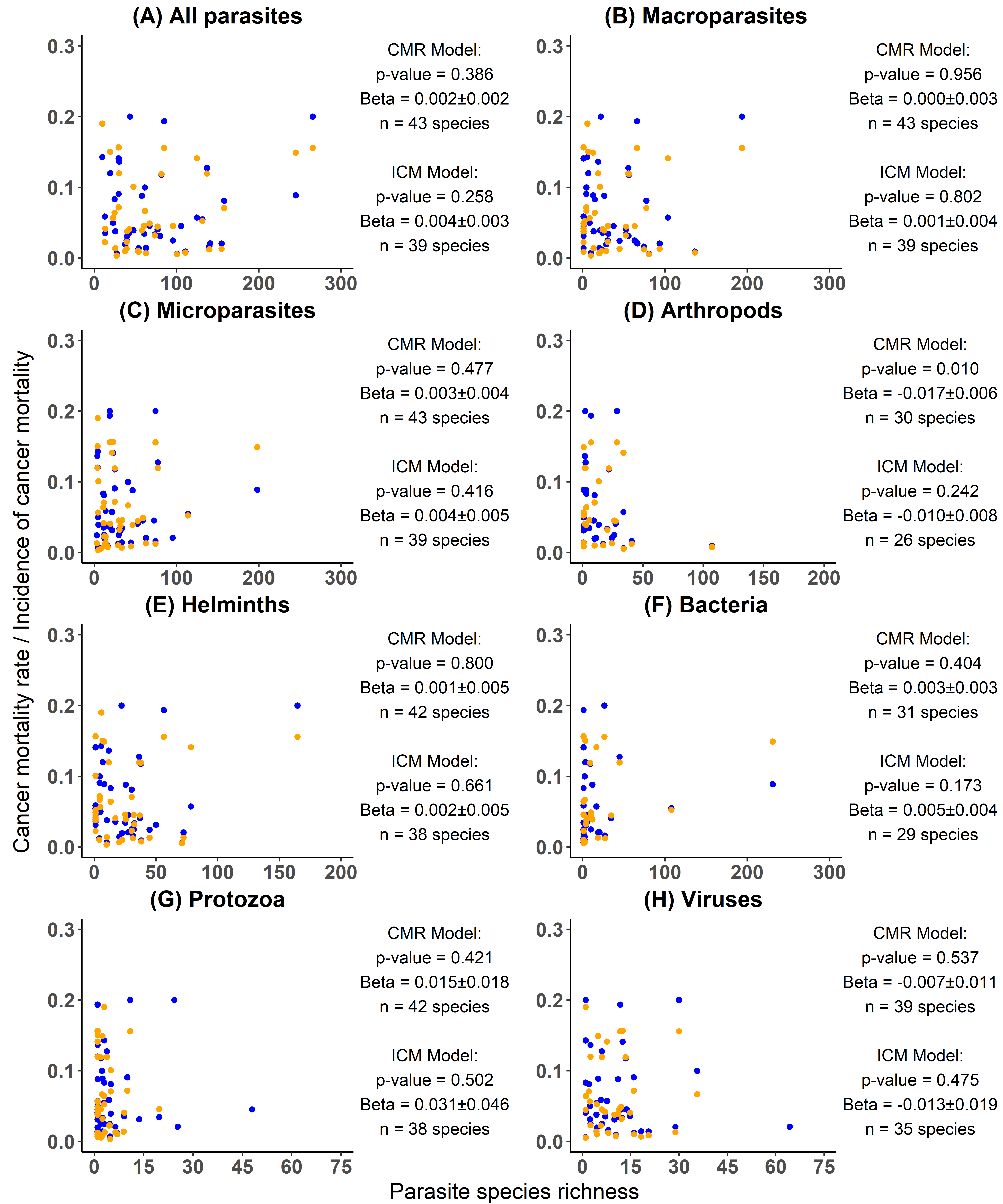
*

**Supplementary Figure 3:** Cancer mortality rate (blue) and incidence of cancer mortality (orange) of captive mammal species as a function of their estimated parasite species richness. The p-value, beta slope between cancer mortality rate/incidence of cancer mortality and parasitic species richness, and the number of species included in the PGLS models are shown for each parasite group. Only mammal species with at least ≥6 publications available in the Global Mammal Parasite Database are included in this analysis.

**
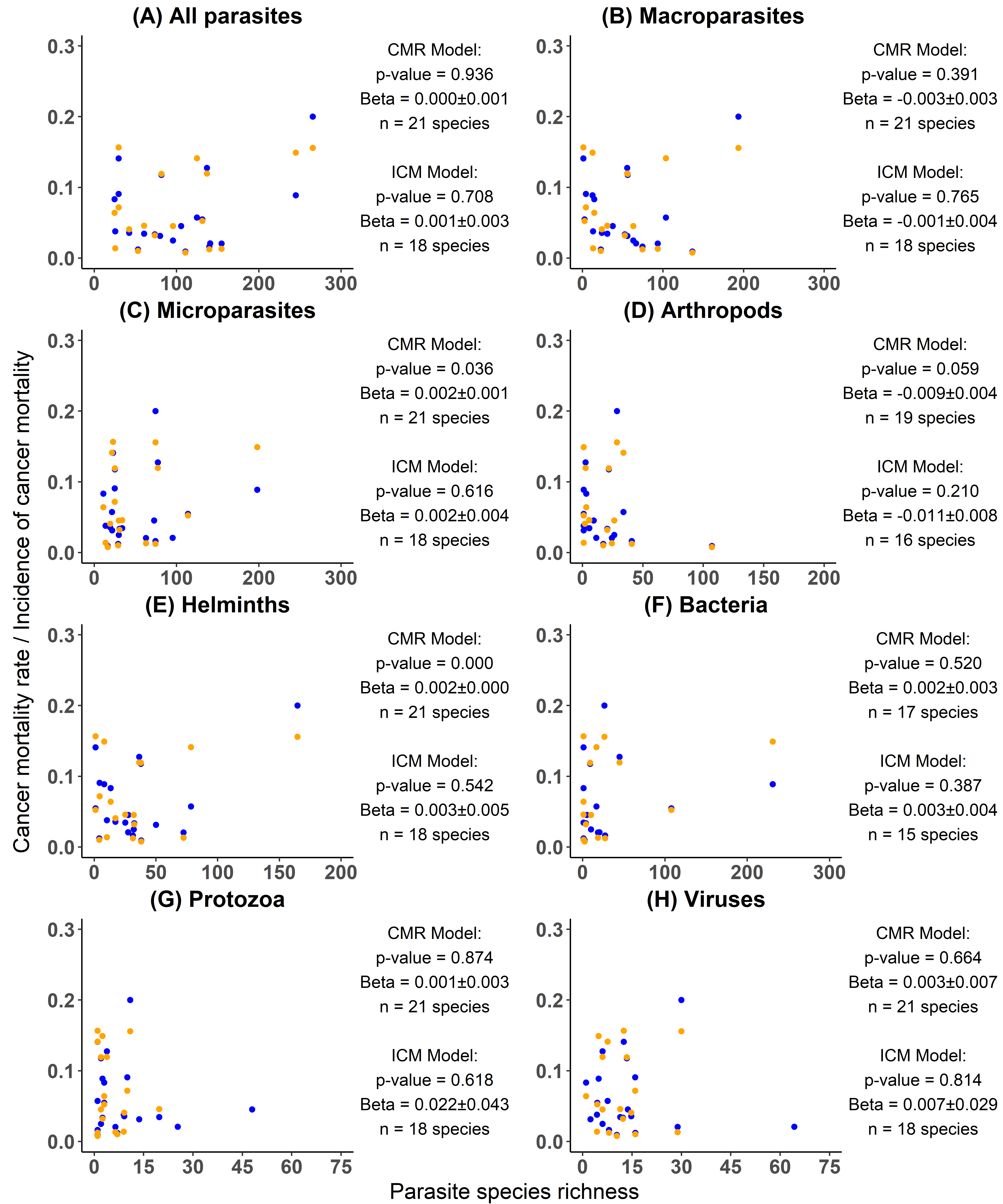
**

**Supplementary Figure 4:** Cancer mortality rate (blue) and incidence of cancer mortality (orange) of captive mammal species as a function of their estimated parasite species richness. The p-value, beta slope between cancer mortality rate/incidence of cancer mortality and parasitic species richness, and the number of species included in the PGLS models are shown for each parasite group. Only mammal species with at least ≥14 publications available in the Global Mammal Parasite Database are included in this analysis.


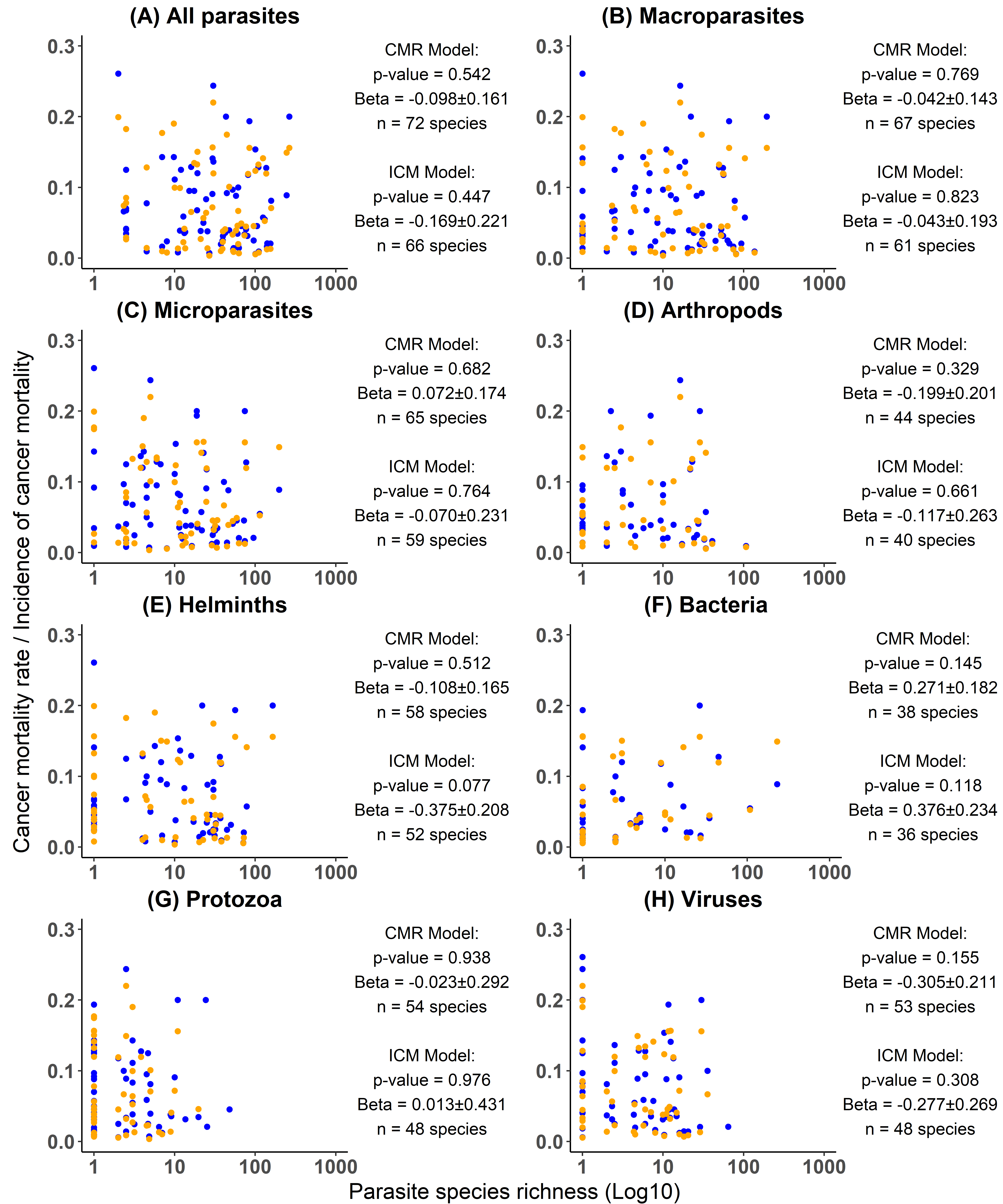


**Supplementary Figure 5:** Cancer mortality rate (blue) and incidence of cancer mortality (orange) of captive mammal species as a function of their estimated parasite species richness (log10 transformed). The p-value, beta slope between cancer mortality rate/incidence of cancer mortality and parasitic species richness, and the number of species included in the PGLS models are shown for each parasite group. Only mammal species with at least ≥2 publications available in the Global Mammal Parasite Database are included in this analysis.


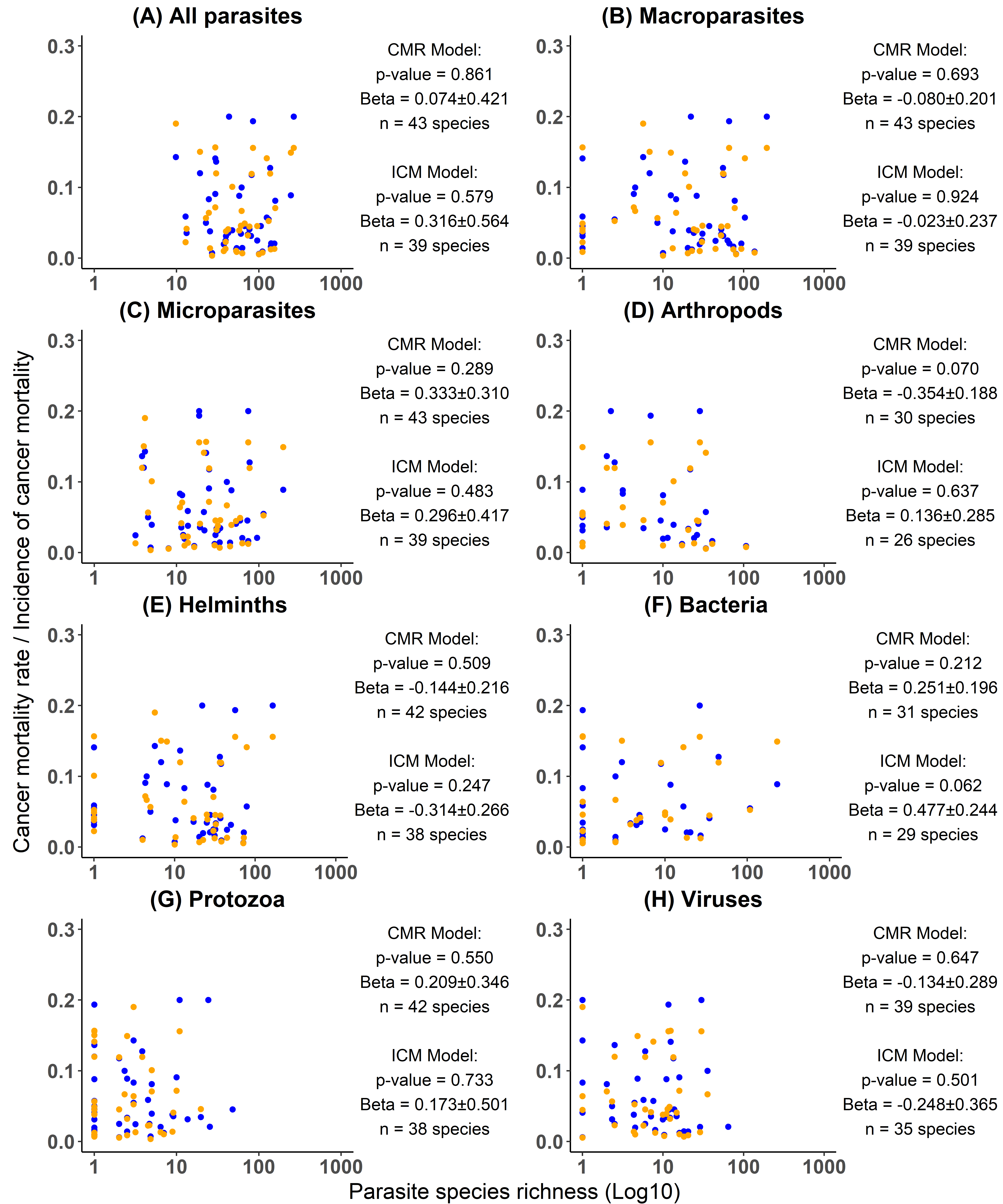


**Supplementary Figure 6:** Cancer mortality rate (blue) and incidence of cancer mortality (orange) of captive mammal species as a function of their estimated parasite species richness (log10 transformed). The p-value, beta slope between cancer mortality rate/incidence of cancer mortality and parasitic species richness, and the number of species included in the PGLS models are shown for each parasite group. Only mammal species with at least ≥6 publications available in the Global Mammal Parasite Database are included in this analysis.


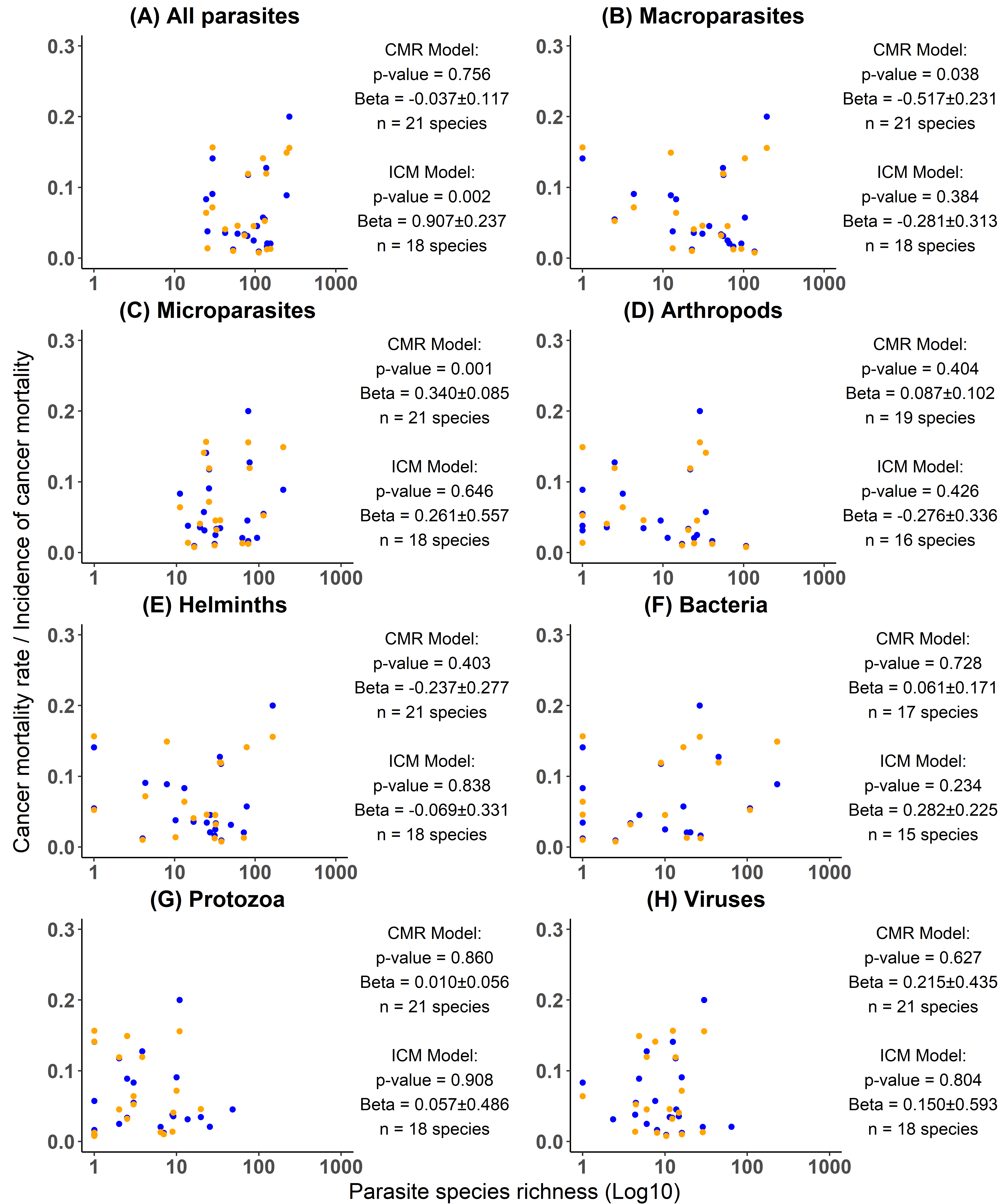


**Supplementary Figure 7:** Cancer mortality rate (blue) and incidence of cancer mortality (orange) of captive mammal species as a function of their estimated parasite species richness (log10 transformed). The p-value, beta slope between cancer mortality rate/incidence of cancer mortality and parasitic species richness, and the number of species included in the PGLS models are shown for each parasite group. Only mammal species with at least ≥14 publications available in the Global Mammal Parasite Database are included in this analysis.
